# Design of a Smart Ultrasonic Transducer for Interconnecting Machine Applications

**DOI:** 10.3390/s90604986

**Published:** 2009-06-24

**Authors:** Tian-Hong Yan, Wei Wang, Xue-Dong Chen, Qing Li, Chang Xu

**Affiliations:** 1 College of Mechatronics Engineering, China Ji Liang University, Xueyuan Road, Xiasha University Park, Hangzhou 310018, China; E-Mails: thyan@163.com (T.H.Y.); wangwei@cjlu.edu.cn (W.W.); xuchang@mail.hj.zj.cn (C.X.); 2 National Key Lab for Digital Manufacturing and Equipment Lab, Huazhong University of Science & Technology, Wuhan 430074, China; E-Mail: chenxd@mail.hust.edu.cn

**Keywords:** ultrasonic transducer, wire bonding, axial / longitudinal vibration mode

## Abstract

A high-frequency ultrasonic transducer for copper or gold wire bonding has been designed, analyzed, prototyped and tested. Modeling techniques were used in the design phase and a practical design procedure was established and used. The transducer was decomposed into its elementary components. For each component, an initial design was obtained with simulations using a finite elements model (FEM). Simulated ultrasonic modules were built and characterized experimentally through the Laser Doppler Vibrometer (LDV) and electrical resonance spectra. Compared with experimental data, the FEM could be iteratively adjusted and updated. Having achieved a remarkably highly-predictive FEM of the whole transducer, the design parameters could be tuned for the desired applications, then the transducer is fixed on the wire bonder with a complete holder clamping was calculated by the FEM. The approach to mount ultrasonic transducers on wire bonding machines also is of major importance for wire bonding in modern electronic packaging. The presented method can lead to obtaining a nearly complete decoupling clamper design of the transducer to the wire bonder.

## 1. Introduction

The rapid evolution in microelectronic technology is a well known phenomenon. IC packaging is developing very fast to meet the key market demand for smaller electronic products; the gap between the IC technology and the equipment technology needs to be bridged. Wire bonding is the most commonly used interconnection technology in the microelectronics manufacturing industry [1,2]. In 2010 and beyond, high density packaging is expected to move from today's asymmetric packaging to a highly integrated and symmetric concept involving the capability of interconnecting NANO-IC, so high-speed and high-precision wire bonding machines are urgently needed stringently. Ultrasonic vibratory energy plays a key role in the ultrasonic bonding process. The ultrasonic system of a wire bonder consists of an ultrasonic generator and transducer. The generator provides electrical power to the transducer at a given frequency, which is not the subject of this article. Constant power and time bonding is a common practice, but not the best option. In reality each bonding phase requires application of a different level of energy. The risk is that a large number of bonds may receive too much energy and thus be “overbonded”, resulting in a significant quality loss. Chip tilting and silicon cratering were compared for the two modes of thermosonic flip-chip bonding. Finite element model (FEM) using ANSYS^®^ was used to study the effect of rigidity of transducer and the stress induced on the silicon layer during bonding [3]. There are three problem areas in ultrasonic bonding: bonding energy transform, bond process control and bond quality monitoring. The main concern in wire bonding technology is the difficulty in transducer design, monitoring and influencing the bond quality during the bond process, and theories and attempts to solve this problem are as old as the technology itself [4-9]. In the age zero defect manufacturing, electronics manufacturing engineers are seeking the best solution based on more reliable statistical data and analysis on bonding dynamics [10-15], e.g. the sensor configuration and properties had been studied in [10], the modeling and simulation for a transducer with flange constraints had been presented in [13], the characteristics of the longitudinal-complex transverse bonding system was studied by [14] and the piezocomposite driver for transducer had been mainly investigated by Or *et al.* [15]; they have all contributed to obtaining a best solution for system design.

It is universally acknowledged that when ultrasonic energy removes brittle surface oxide in the beginning phase of bonding processes, the structure of the crystal lattice on the new bare metal surface is incomplete, then one material atom begins to transfer another material. Some bonder manufacturers have suggested that the cleaning phase requires more power than the mixing phase. The bondability as a function of the ultrasonic power-vs-time profile needs to be studied. The best built-in sensor for ultrasonic vibratory energy will work for monitoring the ultrasonic power directly. The objective of this research was to understand the bond process by monitoring the effect of input power on its performance. Eleven groups of bonding data at different energy level settings (both successful and unsuccessful) were studied seeking a relation between bondability window and input power. A Laser Doppler Vibrometer was used to record the structural response and to explain the phenomena occurring in the experiments [16-19]. The high vibration frequency of the transducer is proven by process tests to be effective to achieve robustness and to improve the ball roundness [16,17]. This not only benefits the fine pitch of wire bonding capabilities, but also decreases the minimum wire bonding temperature required for the applied bonding force. Currently, the longitudinal working frequency is up to 200 kHz [20]. This paper presents a modeling procedure for a transducer assembly by using FEM and investigated how the assembly is constrained, however, this modeling method is not a scientific contribution by itself, as development of transducers is largely based on trial-and-error, but by seeing how the initial parameters are iteratively defined and modified according to experimental results, through the presented procedure, the readers can design their needed ultrasonic transducers. The remainder of this paper is organized as follows. Section 2 gives a brief introduction to the wire bonding procedure and the function of transducer systems. In Section 3, the analytical modeling techniques and the iteration design method for the transducer are presented. In Section 4, the FEM simulation results are presented and discussed. Finally, the conclusions are drawn in Section 5.

## 2. The System Configuration and Performances

**Figure 1.**
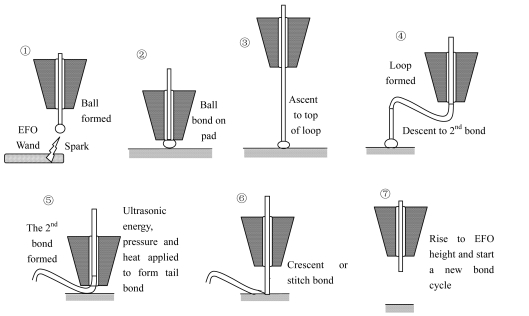
Simplified procedure for making a ball-stitch wire interconnection with a capillary.

Wire bonding is the most commonly used interconnect technology in the microelectronics manufacturing industry [2]. In this interconnection method, bonding wires carry power and signals between the active semiconductor circuits and the lead frame or substrate metallization. Gold wire is usually used because of its easy handling and strong bond with the bond pad metal. Shown in Figure 1 are the steps involved in thermosonic bonding.

The main steps are as follows [2]: (1) Gold wire is threaded through the capillary and electric flame-off (EFO) is used to form a ball on the end of the wire. (2) The capillary descends and presses the gold ball onto an aluminum terminal set on the surface of an IC chip or die. (3) Ultrasonic bursts of energy are applied with the capillary, creating a weld using atomic interdiffusion between the gold ball and bonding pad. (4) The capillary ascends vertically to play out sufficient wire to form a loop as it moves toward the second bond site. (5) The capillary descends to make the second bond (crescent bond) onto the substrate or leadframe with ultrasonic energy, pressure and heat applied. (6) The wire clamp is closed and the capillary moves vertically to break the wire at the heel of the second bond. (7) The capillary rises to EFO height and can start a new bond cycle. Once a bonding cycle is completed, a precisely shaped wire connection called a wire loop is created as the capillary descends to a target position for the second bond.

**Figure 2.**
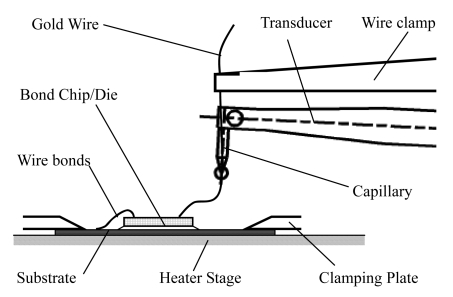
The schematic depiction of electrical interconnect packaging of wire bonder.

**Figure 3.**
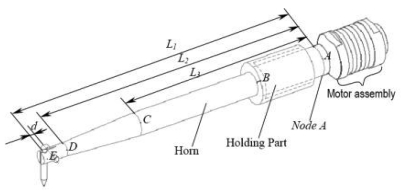
The geometry of the wire bonder transducer assembly.

The schematic setup for electrical interconnect packaging is shown through a schematic drawing in Figure 2. Elastic gold wires were soldered to an IC chip/die or printed circuit board (PCB). The chip or PCB was clamped via clamping plates. Wires were soldered to the chip or PCB and connected to the substrate or leadframe. During automatic loading of the substrate, the clamping plate is in an upper position. As soon as the bonding chip or PCB has reached the bonding area, the clamping plate moves to the lower position in order to clamp the substrate to the heater stage, as shown in Figure 2. Simultaneously, the alignment process will be prepared after the loading is finished. After alignment, the contacting force and bonding process will be taught, then the wire bonding would begin. The commercially available transducer used in whole bond head assembly is shown in Figure 3.

## 3. Finite Element Modeling Techniques for the Transducer

### 3.1. Parts Models and Design Rules

The design for the ultrasonic transducer should including the following main parts:

#### 1. Ultrasonic motor assembly

The motor assembly, also called a converter, sometimes includes several pre-stressed piezoelectric rings between two end-masses. In this part the most suitable piezoelectric material must be selected and the pre-stress between the piezoelectric rings must be defined. Based on the previous product design, experiences and the experimental results, the material elastic constants and the friction sources must be identified and quantified. According to the repeated calculating verifications, it can be found that these parameters would not affect the resonant frequency sensitively, but they are sensitive to response amplitudes. Possible undesired nearby modes must be removed. The front plate is titanium and the back plate is aluminum.

#### 2. Ultrasonic sonotrode horn

A cylinder-shaped sonotrode is connected to the motor assembly, in order to transmit the ultrasonic energy. Being characterized by a lower thermal expansion coefficient, the use of titanium, steel, and aluminum as horn materials are proven by process tests to be effective in usage of the wire bonder. In this phase the shape must be determined; usually these parameters are determined based on the previous design and rule of thumb. Here the shape was chosen as coned-cylinder, the main vibration mode and the parasitic modes must be found and analyzed for dimensions.

#### 3. Ultrasonic sonotrode with amplifie

A particular geometry, in our case a conical cylinder, is used near the top part of the main cylinder in order to amplify the ultrasonic amplitude. In this phase the geometry of the amplifier must be tuned with the required frequency and amplitude. A high vibration frequency of the transducer (now up to 138 kHz or 200 kHz) was proven by process tests to be effective for the robustness and to improve the ball roundness. This is not only to benefit the fine pitch of wire bonding capabilities, but also to decrease the minimum of wire bonding temperature with the applied bonding force. Though Al and steel can be used for the horn part, titanium was selected for horn design and analysis finally due to its special properties [17], as shown in Table 1.

#### 4. Transducer capillary

The capillary fixed at the tip of the amplifier is also introduced in the simulation model, in order to account for integrality of the transducer assembly.

#### 5. Transducer Holder

The holding part is used for mounting the transducer assembly in the wire bonders, there are two types of boundary conditions in design: a) The holder part is introduced onto the body of the transducer without constraints. In this case, the position of the holder would be calculated and set at a node of the ultrasonic field; b) Constrained holder. The holder is fixed in some states, corresponding to the screws fixing the transducer onto the wire bonder. The affection of constrained state and the disturbances in the ultrasonic field created by the mounting must be determined and carefully quantified.

Figure 3 shows the simulated mechanical geometry of the ultrasonic wire bonder transducer, indicated in the figure with the corresponding design part. The properties for various materials used in the analysis are listed in the following Table 1.

### 3.2. FEM Simulation of the Transducer

The initial design of each part is obtained and verified through FEM simulations. The simulated ultrasonic transducer is then built and characterized experimentally through the Laser Doppler Vibrometer (LDV) and Agilent 4294A impedance analyzer and spectrum analyzer. The impedance and electrical resonance spectra are measured. Then the comparison of simulated results with experimental data allows the parameters updating of FEM models, in particular the damping coefficients and constraints interfaces, to be iteratively tuned and updated in a set of discrete values. For the build-up of a complex shape system, it is usually too difficult to solve the optimal value through a mathematical optimizing procedure, so it's different from solving the mathematical optimization; for instance, sizing or topology optimization [21]. Finally the achieved FEM simulations could exhibit an accurate prediction on the vibration behavior of the ultrasonic modules, then the FEM could be used for other geometry dimensions adjusting on obtaining the useful higher longitudinal modes.

The resonant frequencies and vibration mode shapes of the whole ultrasonic transducer were computed using ANSYS. With the piezoelectric effect of motor assembly being neglected, the mechanical FEM model was used. This FEM model enables the designer first of all to find out the vibration modes that can be excited in a given frequency range and to characterize accurately the displacement need in application. The frequency responses give the information on all modes, which exhibit the significant amplitude. Moreover, small holes and fillets in the concentrator horn and screws for mounting the capillary are ignored because of difficulty in meshing and computing. All parts in the assembly are assumed to have perfect mechanical coupling to horn. Fixed boundaries assumed on the cylindrical surface of the holder and Points clamping are carried respectively.

## 4. FEM Results and Discussion

**Figure 4.**
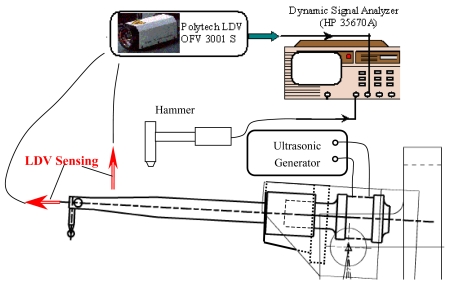
The experimental transducer testing system for FE model calibration.

The first two resonance modes are computed and compared to the frequency response functions and impedance spectrum. Inertia and frequency calculations are based on the three following conditions:

- Transducer tip is maintained at a distance of 88.6 mm away from the pivot center;
- Fixing locations are kept same/constant for all designs;
- In cylindrical holder design, the transducer is fixed at eight location points or completely clamped. The ideal holding point for the transducer should be a node located at point *B* when working at a longitudinal mode for transforming the mechanical energy at a certain frequency, e.g. 95 kHz or 138 kHz. Whereas in barrel holder design, the two dimensions for its diameter are considered for FEM simulation and experimental testing, one diameter is 13.0 mm, the other diameter is 11.0 mm; the studied cases and final dimensions are shown in Table 2. The FE Model for a commercial transducer was calibrated by experiments results, which are obtained from the setup shown in Figure 4.
- If joint stiffness in system level is considered, these frequencies would be less and tip deflection would be higher, the additional joint stiffness should be possibly avoided in design.

**Figure 5.**
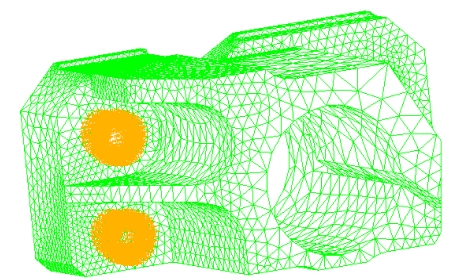
The Geometry and its FEM of horn holder's clamper.

**Figure 6.**
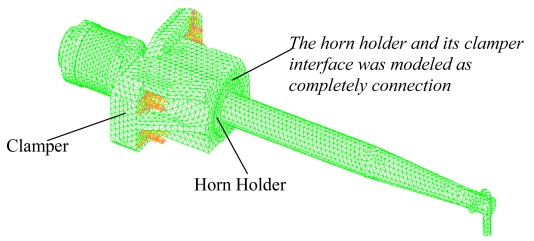
The transducer model with holder constrained through clamper fixed with four screws.

**Figure 7.**
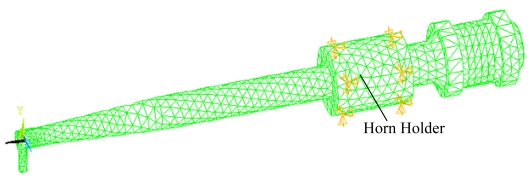
The transducer model with holder constrained by eight points.

The motor assembly transforms the electrical energy into mechanical energy through the ultrasonic motor made up of piezoelectric rings. Here the piezoelectric effect of the motor assembly is neglected to understand the dynamic characteristics of the compact geometry, it was shown that the motor of the motor can be characterized by few radial vibration modes [13]. The connection of horn holder and clamper was modeled as completely connected and the clamper was fixed through four screws holes, as shown in Figure 5. The whole model is shown in Figure 6. The model for horn holder fixed directly via eight points (Cases 2 and 4 in Table 2) is shown in Figure 7.

With horn holder diameter 13.0 mm, from the experimental results for transducer horn completely held by clamp and fixed by four screws, it can be seen the resonant frequencies of Mode 1 and Mode 2 in the concerned low frequency range are 1132 Hz and 1332 Hz respectively. The results are nearly the same with capillary clamped vertically and horizontally. For the case of horn completely clamped via horn holder and fixed via four screws, the comparison between the FEM simulation and experiments is in Table 3.

**Figure 8.**
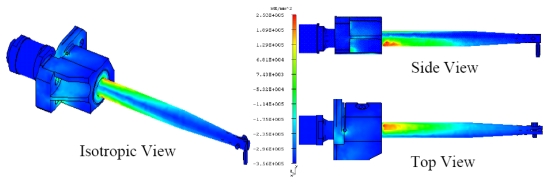
The stress distribution under mode 1 vibration predicted by FEM method.

It can be seen clearly that they agree very well. FEM is very accurate. Through the mode shape by FEM simulation, other information can also be clearly seen, for example, the corresponding stress distribution of each mode. Figure 8 shows the stress distribution of mode at 1,140 Hz, obviously it is a bending mode, the lower modes are useless and harmful for the system itself, only longitudinal modes can be used for electronic packaging.

**Figure 9.**
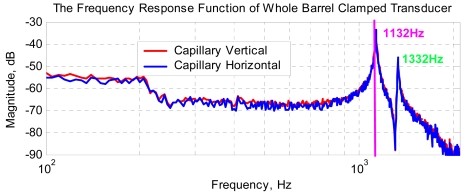

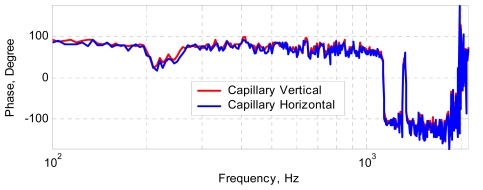
The measured frequency responses of transducer with complete clamping at low frequency range (horn diameter: 13.0 mm, and fixed by four screws on clamper).

Two typical experimental frequency response functions at low frequency range are shown in Figure 9.

**Figure 10.**
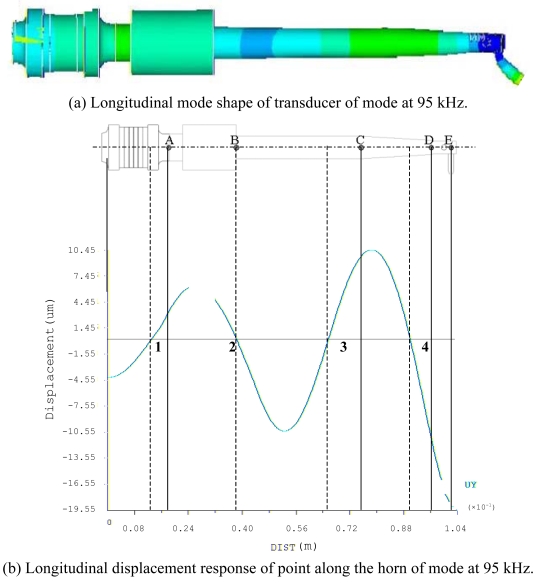
Useful longitutinal mode of transducer with horn holder completely clamped and fixed by four screws on clamper at low frequency (diameter 13.0 mm). (a) Longitudinal mode shape of transducer of mode at 95 kHz. (b) Longitudinal displacement response of point along the horn of mode at 95 kHz.

**Figure 11.**
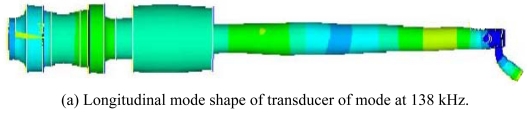

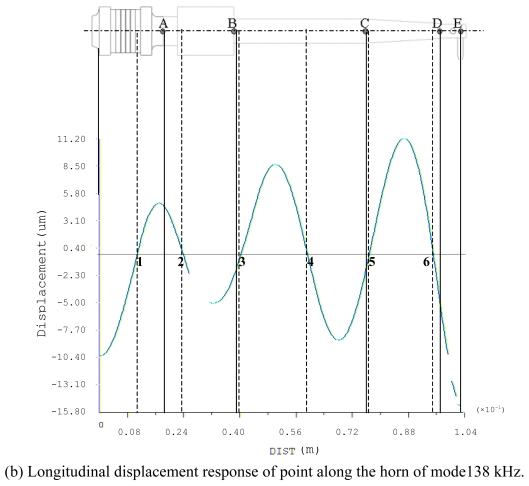
Useful longitutinal mode of transducer with horn holder completely clamped and fixed by 4 screws on clamper at high frequency (diameter 13.0 mm). (a) Longitudinal mode shape of transducer of mode at 138 kHz. (b) Longitudinal displacement response of point along the horn of mode138 kHz.

**Figure 12.**
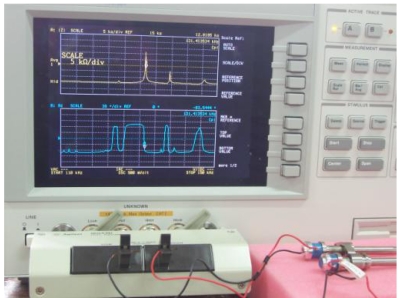
The experiment system for impedance measurement via Agilent 4294 A.

**Figure 13.**
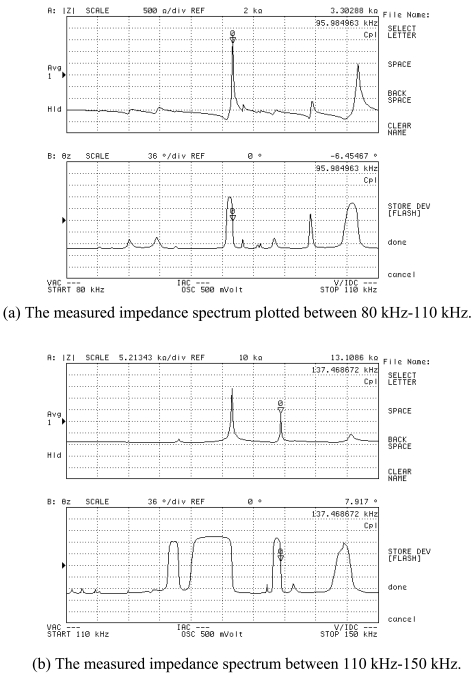
The experimental frequency response functions of the designed ultrasonic transducer with horn diameter 6mm and horn holder diameter 13.0 mm and fixed by four clamp screws. The impedance spectrum is plotted as a function of the frequency between 80 kHz and 150 kHz. (a) The measured impedance spectrum plotted between 80 kHz-110 kHz. (b) The measured impedance spectrum between 110 kHz-150 kHz.

Based on the same modeling techniques, the transducer constraints were changed with horn fixed only by eight points directly to analyze the changes of transducer characteristics. For the inertia and frequency calculation, the transducer is constrained in all directions at the eight clamping locations. It can be seen that both two corresponding modes are improved, i.e. 1,444 Hz and 1,454 Hz, respectively. It means the constraint is much stiffer; this is not good for longitudinal mode displacement transform at high frequency. For the Case 3: the barrel horn holder diameter was decreased to 11.0 mm, the transducer is fixed by four screws through the clamper, the first two resonance modes also decreased a little bit. The results still agree very well with the experimental results. After loosening the screw tightening torque to 0.3 N·m, the resonant frequencies also decreased. For the horn diameter decrease to 11.0 mm, the two lower resonant modes had been increased to 1,540 Hz and 1,652 Hz. Through the results comparison of Table 4, it was found that the clamping state has obvious effects on the dynamic characteristics, the eight points fixed condition is not suitable for mounting the transducer on a wire bonder assembly, the mounting of transducer to machine system through the clamper's four screws is better, but the horn holder diameter has little affect on the vibration characteristics, so this horn holder clamper allows the converter to be attached on the wire bonder not only in axial (longitudinal) nodes but also in radial nodes of the ultrasonic field excited in the horn. Based on this, the useful longitudinal modes of transducer with 13.0 mm horn holder mounted via clamp by four screws were studied numerically in detail. FEM results show that there are a lot of natural frequencies and vibration modes of transducer system within 150 kHz. The vibration mode shapes can be categorized as axial mode, flexural modes, torsion modes, and coupling modes. From the mode shapes, it can be judged that the useful longitudinal modes are 95 kHz and 138 Hz. When driven by electrical signals with appropriate frequency, those vibration modes would be excited for wire bonding usage. The predicted useful longitudinal modes are 95 kHz and 138 kHz, the nearby resonant modes are far from these two modes. The predicted two useful mode shapes and their corresponding displacements along the transducer body are also analyzed in detail, as shown in Figures 10 and 11, respectively. The longitudinal displacement of the transducer tip is 18 μm for the mode at 95 kHz, the displacement for mode 138 kHz can be up to 15 μm, and the displacement can be adjusted through the different driving power for different bonding usages. The impedance of transducer at high frequency range 80∼150 kHz was also measured via the Agilent 4294 A, as shown in Figure 12. It can be seen the main mode frequency remains very clean around 95.8 kHz and 137.5 kHz due to design feature in the assembly, as shown in Figures 13(a, b). It can be seen that the FEM predicted results are also quite convincing after updated at low frequency range by modal testing results, the higher useful longitudinal modes are agree very well.

## 5. Conclusions

The resonance characteristics of an ultrasonic transducer system used in our wire bonding system was found to be quite complicated when measured by an impedance analyzer. The Laser Doppler Vibrometer (LDV) was used to measure the transducer vibration displacement distributions, and standing wave patterns were found along the surface of the concentrator. The FEM of transducer system was set up, and the frequencies of the first 100 allowable modes were computed. Based on the previous experience and model updating via the experimental results, the first several resonant modes of the transducer agreed well with the value measured by LDV method. Then further study was carried out. It was found that the axial mode should be the dominant mode of the transducer. Undesirable non-axial modes, especially higher order flexural modes, also were found to occur in the transducer operating frequency range and are difficult to completely eliminate. The displacement distributions along the horn in longitudinal mode, therefore, are the resultant displacement of all the allowable modes being excited in the transducer. However, the excitations of these deleterious modes were found to be small enough, and good bonds can still be formed. The results obtained in this work have been used in finding an appropriate and optimal transducer that satisfies the following wire bonding application. The transducer which has newly desired high-frequency ultrasonic mode can be designed, analyzed, prototyped and tested via the presented modeling method and especially modeling parameters for interfaces of the whole assembly. The mode shapes and displacement of the transducer at ultrasonic frequencies of 95.98 kHz and 137.5 kHz have been analyzed, respectively. The longitudinal vibration modes are also plotted out to show the nodal locations. The transducer is made of titanium and vibrates at the resonance frequency of 95.98 kHz and 137.5 kHz. The new transducer is completely fixed on the wire bonder with a barrel holder whose special geometry was calculated by means of FEM simulations. This barrel holder allows the converter to be attached on the wire bonder not only in longitudinal nodes but also in radial nodes of the ultrasonic field excited in the horn. This leads to a total decoupling of the transducer to the wire bonder. The approach to mounting ultrasonic transducers on an electronic packaging machine is of major importance for modern wire bonding.

## Figures and Tables

**Table 1.** Material properties chosen for the model.

	**(Unit)**	**Steel**	**Titanium**	**Alumina**	**PZT**	**Aluminum**	**Capillary**

Young's Modulus	Gpa	210	110	300	92	70	301
Poisson's ratio	-	0.3	0.32	0.2	0.33	0.30	0.21
Density	Kg/m^3^	7800	4430	3720	7700	2700	3718
Thermal Expansion	10^-6^/K	15	9	-	d31,d32*	23	-

* d31 = d32 = 166×10^-12^ mV^-2^

**Table 2.** The studied transducer assembly cases.

**Length (mm)**	**Horn Diameter**	**Holder Completely Clamped, fixed via four screws**	**Holder fixed via eight points directly**
*L_1_* = 82.0; *L_2_* = 75.0 *L_3_* = 54.0; *d* = 2.0	13.0 mm	Case 1	Case 2
	11.0 mm	Case 3	Case 4

**Table 3.** Comparison of results for transducer with steel horn (holder diameter 13.0 mm).

**Experimental Results for Complete Holder Clamping, fixed four screws Torque = 0.4 N·m**			**Case 1: FEA with Complete Holder Clamping**		**Case 2: FEA Using 8 Points Constraints**	
	Capillary Vertical (Hz)	Capillary Horizontal (Hz)	Mode 1 (Hz)	Mode 2 (Hz)	Mode 1 (Hz)	Mode 2 (Hz)
Mode 1	1,132	1,132	1,140	1,331	1,444	1,454
Mode 2	1,332	1,332	+0.44%	-0.3%	+27%	+9.0%

**Table 4.** Comparison of results for transducer with steel horn (holder diameter 11.0 mm).

**Experimental Results for Complete Holder Clamping, fixed four screws**				**Case 3: FEA Using Complete Holder**		**Case 4: FEA Using 8 Points Constraints**	
Torque = 0.5 N·m		Torque = 0.4 N·m					
Capillary Vertical (Hz)	Capillary Horizontal (Hz)	Capillary Vertical (Hz)	Capillary Horizontal (Hz)	Mode 1 (Hz)	Mode 2 (Hz)	Mode 1 (Hz)	Mode 2 (Hz)
1,100	1,065	1,080	1,045	1,123	1,295	1,540	1,652
1,333	1,280	1,333	1,276	+2.1%	-2.8%	+40%	+24%

## References

[1] Tummala R.R., Rymaszewski E.J. (1997). Microelectronics Packaging Handbook [M].

[2] Harman G. (1997). Wire Bonding in Microelectronics: Materials, Processes, Reliability, and Yield.

[3] Leung M.L.H., Lai-Wah H.C., Chou-Kee P.L. Comparison of bonding defects for longitudinal and transverse thermosonic flip-chip.

[4] Or S.W., Chan H.L.W., Lo V.C., Yuen C.W. (1998). Ultrasonic wire-bond quality monitoring using piezoelectric sensor. Sens. Actuat..

[5] Ramminger S., Seliger N., Wachutka G. (2000). Reliability model for Al wire bonds subjected to heel crack failures. Microelectron. Reliab..

[6] Mayer M. (2000). Microelectronic bonding process monitoring by integrated sensors..

[7] Chiu S.S., Chan H.L.W., Or S.W., Cheung Y.M., Liu P.C.K. (2003). Effect of electrode pattern on the outputs of piezosensors for wire bonding process control. Mater. Sci. Eng..

[8] Moorhouse A.T., Gibbs B.M. (1993). Prediction of the structureborne noise emission of machine: development of a methodology. J. Sound Vib..

[9] Han L., Zhong J., Gao G.Z. (2008). Effect of tightening torque on transducer dynamics and bond strength in wire bonding. Sens. Actuat. A.

[10] Chua P.W.P., Li H.L., Chan H.L.W., Ng K.M.W., Liu P.C.K. (2002). Smart ultrasonic transducer for wire-bonding applications. Mat. Chem. Phys..

[11] Chua P.W.P., Chong C.P., Chan H.L.W., Ng K.M.W., Liu P.C.K. (2003). Placement of piezoelectric ceramic sensors in ultrasonic wire-bonding transducers. Microelectron. Eng..

[12] Or S.W., Chan H.L.W. (1998). Dynamics of an ultrasonic transducer used for wire bonding. IEEE Trans. Ultrason. Ferroelectr. Freq. Control.

[13] Parrini L. (2003). New technology for the design of advanced ultrasonic transducers for high-power applications. Ultrasonics.

[14] Tsujino J., Yoshihara H., Sano T., Ihara S. (2000). High-frequency ultrasonic wire bonding systems. Ultrasonics.

[15] Or S.W., Chan H.L.W., Liu P.C.K. (2007). Piezocomposite ultrasonic transducer for high-frequency wire-bonding of microelectronics devices. Sens. Actuat. A.

[16] Michael M., Juerg S., Daniel B., Oliver P., Juergen S. (2001). Active Test Chips for in situ Wire Bonding Process Characterisation.

[17] Parrini L. (2002). Advances process characterization for 125 kHz wire bonder ultrasonic transducer. IEEE Trans. Compon. Packag. Technol..

[18] Chylak B., Qin I.W., Eder J. (2004). Achieve Optimal Wire Bonding Performance through Ultrasonic System Improvement. Kulicke and Soffa Industries, Inc. SEMICON® Singapore..

[19] Ditri J., Eder J. (2002). Real Time Ultrasonic Bond Quality Monitoring.

[20] UTHE 200 kHz SYSTEM TRANSDUCERS http://www.bita.se/bitase1/u-200k.htm.

[21] Haftka R.T., Gürdal Z., Kamat M.P. (1990). Elements of Structural Optimization.

